# *Candida auris* in the burn unit: a prolonged biphasic outbreak

**DOI:** 10.1017/ash.2024.74

**Published:** 2024-05-27

**Authors:** Laura Pedersen, Jake Mikesell, Robert Marcaccini, Connie Atkinson, Kaila Cooper, Michael Feldman, Barry Rittman, Patrick Ching, Alexandra Bryson, Michelle Doll

**Affiliations:** Virginia Commonwealth University, Richmond, VA, USA

## Abstract

*Candida auris* is an emerging pathogen responsible for healthcare-associated infections and outbreaks. This organism has a high tolerance to both high temperatures and high salinity. We describe our experience with a *C. auris* outbreak in an 8-bed inpatient burn unit at an academic medical center.

## Introduction

*Candida auris* is an emerging pathogen with a propensity to cause life-threatening infections in vulnerable patients.^1,2^ We describe a biphasic *Candida auris* outbreak on our 8-bed inpatient burn unit over 8 months.

## Methods

A *Candida auris* outbreak occurred in our Surgical Trauma Intensive Care Unit (STICU) in early 2023. Within one month, *C. auris* was identified in the Burn ICU (BICU). The facility is an 865-bed tertiary care center and ICUs are on different floors. The same providers work in both units. During this period, no patients were transferred from the STICU to the BICU. The clusters of *C. auris* occurred in the context of regional outbreaks of *C. auris* in other hospitals and post-acute care facilities.

The index patient in the BICU was identified when a tissue culture grew *C. auris*. Since the index patient had a prolonged hospitalization, point prevalence testing (PPT) was conducted. Testing was performed by skin swab of the bilateral axilla and groin with specimens sent to the health department for reverse transcription-polymerase chain reaction (RT-PCR) testing for *C. auris.* Testing was requested for all patients in the BICU weekly for 4 weeks and identified 1 colonized patient.^2^ The PPT in the BICU was stopped when two consecutive weeks were negative. The cluster was considered resolved, and both patients were discharged. The STICU had a simultaneous outbreak, overlapping with phase 1, with 7 patients positive on PPT which resolved before phase two.

A second phase of the outbreak occurred 4 months later when a clinical culture grew *C. auris*.^3^ Prior to testing positive for *C. auris,* patient 3 was under expanded contact precautions (providers changed into clean hospital scrubs for entry) due to the vulnerability of the specialized skin grafts. Point prevalence testing began again in the BICU, with two additional colonized patients^4,5^ identified. Weekly PPT continued in the BICU until two consecutive weeks were negative.

Historically, the BICU used a universal contact precaution strategy with gown and glove use. This was stopped during the COVID-19 Pandemic in early 2020 and not resumed until the second phase of the *C. auris* outbreak.

During the outbreak, infection prevention interventions included: strict contact isolation of all infected or colonized patients with door monitors to enforce adherence, enhanced environmental cleaning of common areas and rooms with a hydrogen-peroxide-peracetic acid combination, which is on the Environmental Protection Agency’s list of P Agents that are active against *C. auris.* The floors were cleaned with detergent. For rooms housing a *C. auris* infected/colonized patient, the floors were cleaned with a hydrogen-peroxide-peracetic acid solution and the floor with detergent. Infection preventionists directly observed terminal cleaning using a hydrogen-peroxide-peracetic acid combination for the room and floors. Cleaning was validated with adenosine triphosphate (ATP) testing before ultraviolet wavelength C (UVC) light treatment with Tru-D^®^ UVC disinfection system and approval for room turnover. If ATP testing failed, the room was re-cleaned until it passed. For terminal cleaning of any isolation room, all supplies in the carts were discarded, carts thoroughly cleaned, and curtains changed.

Seven months after patient #1 was identified, due to the persistent outbreak, environmental samples were collected from the sinks and floor drains of all patient rooms and the procedure room in the BICU using an Eswab^TM^. Cultures were incubated using ChromAgar^TM^ *C. auris* selective media. These areas were cultured because it was hypothesized that *C. auris* can persist in plumbing, similar to gram-negative rods.^3^ Due to limited resources, environmental cultures were restricted to these areas.

### Results/case series

#### Patient 1

This patient presented with >60% total body surface area (TBSA burns), initially treated at an outside center, discharged to a rehabilitation facility, and then admitted to our facility for poor wound healing. The *C. auris* burden at the outside burn center is unknown.

She required multiple procedures, devices, and antimicrobials. On hospital day (HD) 37 a polymicrobial tissue debridement was positive for *C. auris* (Micafungin mean inhibitory concentration (MIC) 0.12 ug/ml, Posaconazole MIC 0.12 ug/ml, Voriconazole MIC 2.0 ug/ml).

She received Posaconazole and then Micafungin for 14 days. On HD132, she was discharged with no known additional infectious complications.

#### Patient 2

Six weeks after patient 1 was admitted, patient 2 was admitted from home with <10% TBSA burns. He required multiple procedures. On HD5, he screened positive for *C. auris* during the first phase of PPT.

The patient was on broad-spectrum antimicrobials and had multiple indwelling devices. He did not require any treatment for *C. auris,* was discharged on HD49, and has not had complications related to *C. auris.*

#### Patient 3

Patient 3 presented from home after sustaining >60% TBSA burn requiring multiple procedures and expanded contact precautions to protect the specialized skin grafts in place. He required prolonged antimicrobials.

Six months after admission, and 4 months after phase one of the outbreak resolved, a polymicrobial wound grew *C. auris* (Posaconazole MIC 0.5 ug/ml, Voriconazole 2.0 ug/ml). He was treated with Micafungin and Posaconazole for two weeks without any additional complications related to *C. auris*.

#### Patient 4

Patient 4 was admitted to the same room previously housing patient 1 after sustaining >3% TBSA burns. On HD30, he screened positive for *C. auris* during phase two PPT. He was on broad-spectrum antibiotics. On HD35, a polymicrobial tissue culture grew *C. auris* (Voriconazole MIC 4.0 ug/ml). He was discharged on 6 weeks of Micafungin for treatment of *C. auris* osteomyelitis without further *C. auris* complications.

#### Patient 5

Patient 5 was admitted with burns to both feet. On HD17, he tested positive for *C. auris* via phase two PPT, while roomed next to patient 4. Before screening, he was on broad-spectrum antibiotics for tissue and bone infections. He underwent multiple debridement procedures. He remains without *C. auris* infection.

### Environmental samples

*C. auris* was detected from the floor drain of the room used by patients 1 and 4. A cover was subsequently placed over the floor drains in all patient rooms of the burn unit.

## Discussion

We describe five patients testing positive for *Candida auris* while hospitalized in a Burn ICU. All patients had multiple risk factors for *C. auris* acquisition.

Patient 3 developed candidiasis approximately two months after the last negative PPT in phase one of the outbreak. Late presentation of *C. auris* infection was concerning for unit transmission despite intensive infection control measures. Delayed colonization despite multiple negative PPTs was reported previously in a burn ICU.^4^ The biphasic timeline suggests an environmental reservoir as reported by other centers.^5^ The drain from the room previously housing patient 1, then patient 4, remained positive for *C. auris* 4 months after discharge and might represent an environmental reservoir. This provides additional evidence that *C. auris* behaves similarly to gram-negative rods in healthcare settings, colonizing water sources and persisting despite cleaning efforts.

We are limited by a lack of access to genomic sequencing but have epidemiologic evidence of unit-acquired colonization and progression to infection despite aggressive infection control measures of enhanced contact isolation and environmental cleaning. Others have reported transmission of *C. auris* in the setting of environmental contamination of the affected patient room despite following recommended cleaning processes.^3^ These findings emphasize the importance of frequent hand hygiene and repeated environmental cleaning to prevent transmission from environmental reservoirs.

We warn institutions that patients can acquire *Candida auris* after initial screening tests are negative and positive patients are discharged. Institutions should consider screening patients in units where *C. auris* is detected, and those being admitted from other healthcare facilities if *C. auris* is present in the region.^6^ Once *C. auris* is detected in a facility, longstanding environmental contamination despite cleaning processes should be assumed until more information on the drivers of biphasic or relapsing outbreaks is available.

## References

[1] Allert S , Schulz D , Kämmer P , Großmann P , Wolf T , Schäuble S , et al. From environmental adaptation to host survival: attributes that mediate pathogenicity of *Candida auris* . Virulence 2022;13:191–214. 35142597 10.1080/21505594.2022.2026037 PMC8837256

[2] Sanyaolu A , Okorie C , Marinkovic A , Abbasi AF , Prakash S , Mangat J , et al. *Candida auris*: an overview of the emerging drug-resistant fungal infection. Infect Chemother 2022;54:236–46. 35794716 10.3947/ic.2022.0008 PMC9259907

[3] Lowe C , Willey B , O’Shaughnessy A , Lee W , Lum M , Pike K , et al. Outbreak of extended-spectrum β-Lactamase–producing *Klebsiella oxytoca* infections associated with contaminated handwashing sinks1. Emerg Infect Dis 2012;18:1242–7. 22841005 10.3201/eid1808.111268 PMC3414015

[4] Alanio A , Snell HM , Cordier C , Desnos-Olivier M , Dellière S , Aissaoui N , et al. First patient-to-patient IntraHospital transmission of clade I *Candida auris* in France revealed after a two-month incubation period. Microbiol Spectr 2022;10:e01833–22. 36094221 10.1128/spectrum.01833-22 PMC9604096

[5] Patterson CA , Wyncoll D , Patel A , Ceesay Y , Newsholme W , Chand M , et al. Cloth lanyards as a source of intermittent transmission of *Candida auris* on an ICU*. Crit Care Med;49:697–701. 33395069 10.1097/CCM.0000000000004843

[6] Centers for Disease Control and Prevention. Screening for *Candida auris* colonization in healthcare settings [Internet]. 2024 [cited 2024 Feb 29]. https://www.cdc.gov/fungal/candida-auris/c-auris-screening.html

